# An in-flight respiratory emergency and survival in the sky

**DOI:** 10.4103/0974-2700.66559

**Published:** 2010

**Authors:** S SenthilKumaran, P Thirumalaikolundusubramanian

**Affiliations:** Sri Gokulam Hospital and Research Institute, Salem, Trichy, India; 1 Chennai Medical College Hospital and Research Centre, Irungalur, Trichy, India

Sir,

Air travel has now become a common mode of transport. A variety of low-cost carriers have made air travel accessible to a larger section of the Indian population. As a result of this, nearly 15 million people travel on domestic airlines each year in India.[1] In general, air travel can precipitate or contribute to medical problems in a number of ways, in previously healthy travelers also.[2] The exact number of medical emergencies on board is not well known because airlines are not required to report these occurrences or any flight diversions due to medical problems. The incidence of significant in-flight emergencies is approximately one per 10,000–40,000 passengers, with one death occurring per 3–5 million passengers.[3] Although medical emergencies in air are generally rare, the vast majority are likely due to exacerbation of pre-existing medical problems. Respiratory problems were the most common in view of reduced cabin pressure and low humidity, predisposing to the theoretical risk of bronchospasm as a result of water loss from the bronchial mucosa. Fifty percent of these cases were related to asthma and one-third of them were due to forgotten medication.[4] The present report deals with the difficulties encountered while treating an in–flight respiratory medical emergency and utilization of locally available material to tackle the crises.

A 24-year-old male started complaining of shortness of breath and chest discomfort 1 h after the flight took off from the airport. He was immediately given oxygen supplement (4 L/min via a face mask) by the air hostess and she called for help from other crew members. The captain paged for medical assistance on board. The first author being an emergency physician responded to the call. The copassenger, a first-time air traveler, was a known wheezer and not on regular treatment. He had exacerbations during the winter, which subsided with oral bronchodilators. He was suffering from upper respiratory tract infection of 2 days duration. He denied diabetes mellitus, high blood pressure, heart disease and smoking.

On examination, he was afebrile, dyspnoeic, tachypnoeic (40/min) tachycardic (123 beats per minute) and cyanosed. His blood pressure was 150/70. On auscultation, the lungs were silent and his heart sounds were audible without murmurs. The abdomen was soft and non-tender. A provisional diagnosis of acute exacerbation of bronchial asthma was made. The patient was shifted from the economy class to closer to the emergency exit for want of space in anticipation of cardiopulmonary resuscitation.

Inhaled bronchodilators are one of the most effective therapies for rapid reversal of airway narrowing in acute asthma and, also, have advantages over oral and parenteral routes of delivery. As there was no nebuliser or spacer in the flight, a metered-dose inhaler (MDI) consisting of salbutamol obtained from another copassenger was used. The patient was unable to perform a conventional inhalation maneuver with MDI as he was breathing rapidly.

Specific inhalation techniques are necessary for better bronchodilation. An improper inhaler technique can result in decreased drug delivery and potentially reduced efficacy. Also, he was unable to synchronize inspiration with actuation of the MDI or could hold the breath at the end of inspiration, which was routinely recommended for optimum use. As the patient was in impending respiratory failure and in the austere environment, the other best alternative treatment was to use spacer (one-way valve inhalation devices with repeated tidal breaths), which allows aerosol delivery better than MDI by circumventing the need for the coordinated actuation of the MDI with inhalation.

Moreover, a metaanalysis on bronchodilator delivery in acute airflow obstruction recommends the use of spacer in the treatment of acute airflow obstruction.[5] As there was no spacer available in-flight, a sealed 200 mL plastic bottle containing water was obtained from the air hostess and converted into a spacer locally to deliver the bronchodilator therapy [Figure 1], which helped to deliver the medications with repeated tidal breaths, resulting in a dramatic improvement in the patient. As the patient became stable, he was handed over to the medical team once the flight landed in the airport for further management.

**Figure 1:**
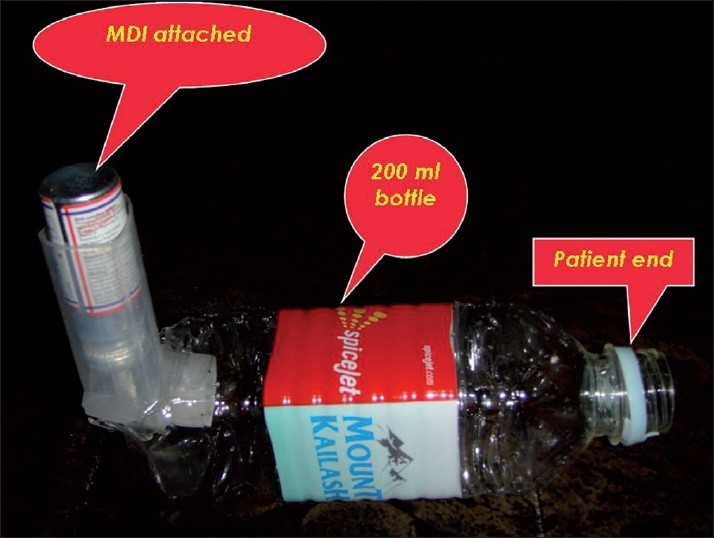
Spacer designed in flight

The rationale behind addition of a spacer allows aerosol delivery by the MDI to be contained in the spacer for a finite period of time. Trapped aerosol that is inhaled for a few seconds after actuation of the inhaler circumvented the need for the coordinated actuation of the MDI with inhalation and prevented the early landing in the nearest airport.

Zar *et al*. have noted the usefulness of modified plastic bottle as an efficient spacer,[6] which produces greater pulmonary aerosol deposition, and have recommended the use of bottle spacers into guidelines for asthma management in developing countries.[7]

The purpose of this communication is, (a) to emphasize the constraints faced by physicians while providing in-flight assistance to copassengers who become ill on board, (b) to create awareness on physiological adaptation to flight, (c) to reorient our medical education and training to face the clinical challenges of in-flight emergencies and on the availability of the resuscitative drugs and devices in commercial flights and (d) to suggest the need for nebuliser on board if permissible or a spacer with MDI and a pulse oximeter in their emergency kit. Let the survival in sky during medical emergencies not be questionable.

## References

[1] http://www.thehindubusinessline.com/2009/11/13/stories/2009111352250100.htm.

[2] Kay RS (1994). Safe air travel. Preventing in-flight medical problems. Nurse Pract.

[3] Urwin A, Ferguson J, McDonald R, Fraser S (2008). A five-year review of ground-to-air emergency medical advice. J Telemed Telecare.

[4] (2002). British Thoracic Society recommendations. Managing passengers with respiratory disease planning air travel. Thorax.

[5] Turner MO, Patel A, Ginsburg S, FitzGerald JM (1997). Bronchodilator delivery in acute airflow obstruction. A meta-analysis. Arch Intern Med.

[6] Zar HJ, Liebenberg M, Weinberg EG, Binns HJ, Mann MD (1998). The efficacy of alternative spacer devices for delivery of aerosol therapy to children with asthma. Ann Trop Paediatr.

[7] Zar HJ, Brown G, Donson H, Brathwaite N, Mann MD, Weinberg EG (1999). Home-made spacers for bronchodilator therapy in children with acute asthma: a randomised trial. Lancet.

